# Supplementary material for: A Parkinson's disease CircRNAs Resource reveals a link between circSLC8A1 and oxidative stress
Source: EMBO Mol Med. 2020 Jul 26;12(9):e11942. doi: 10.15252/emmm.201911942 (PMC7507321; doi:10.15252/emmm.201911942)
Supplement: Supplementary file 1 — Appendix [file EMMM-12-e11942-s001.pdf]

Appendix document

Table of Content:

1. Appendix figure S1: RNA Editing is not correlated to gender and age.

2. Appendix figure S2: No positive correlation between age and circRNA expression in MTG or Amygdala control and PD samples.

3. Appendix figure S3: CircSLC8A1 expression in differentiating ES cells, Ago2-immunoprecipitation validation and miR-128 levels in SN and under oxidation.

Appendix figure S1: RNA Editing is not correlated to gender and age:

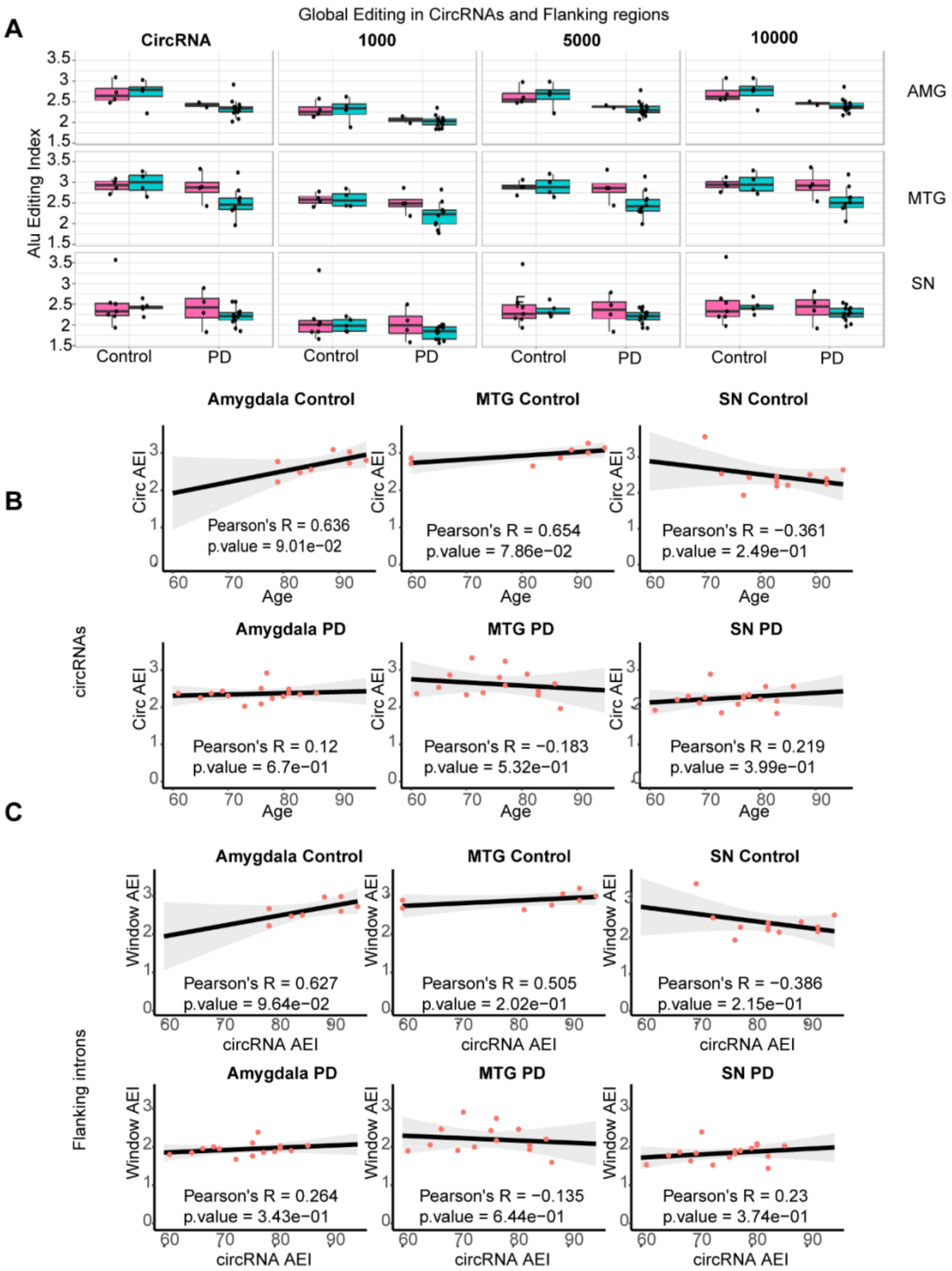

Appendix figure S1: RNA Editing is not correlated to gender and age

(A) RNA editing is not correlated to gender: no statistically significant differences were shown between male & female (T-test p=0.56 for control or 0.26 for PD), female marked in pink and males in cyan. n=8 for Amygdala control, 15 for Amygdala PD, 8 for MTG control and 13 for MTG PD, 10 for SN control and 15 for SN PD. The box is drawn from Q1 to Q3 with a horizontal line drawn in the middle to denote the median. Whiskers mark minimum or maximum values.

(B-C) RNA editing levels are not correlated to age as measured in circRNAs or in the flanking introns: no statistically significant correlation by Wilcoxon test was found between age and AEI in any of the tested groups.

Appendix figure S2: **No positive correlation between age and circRNA expression in MTG or Amygdala control and PD samples**

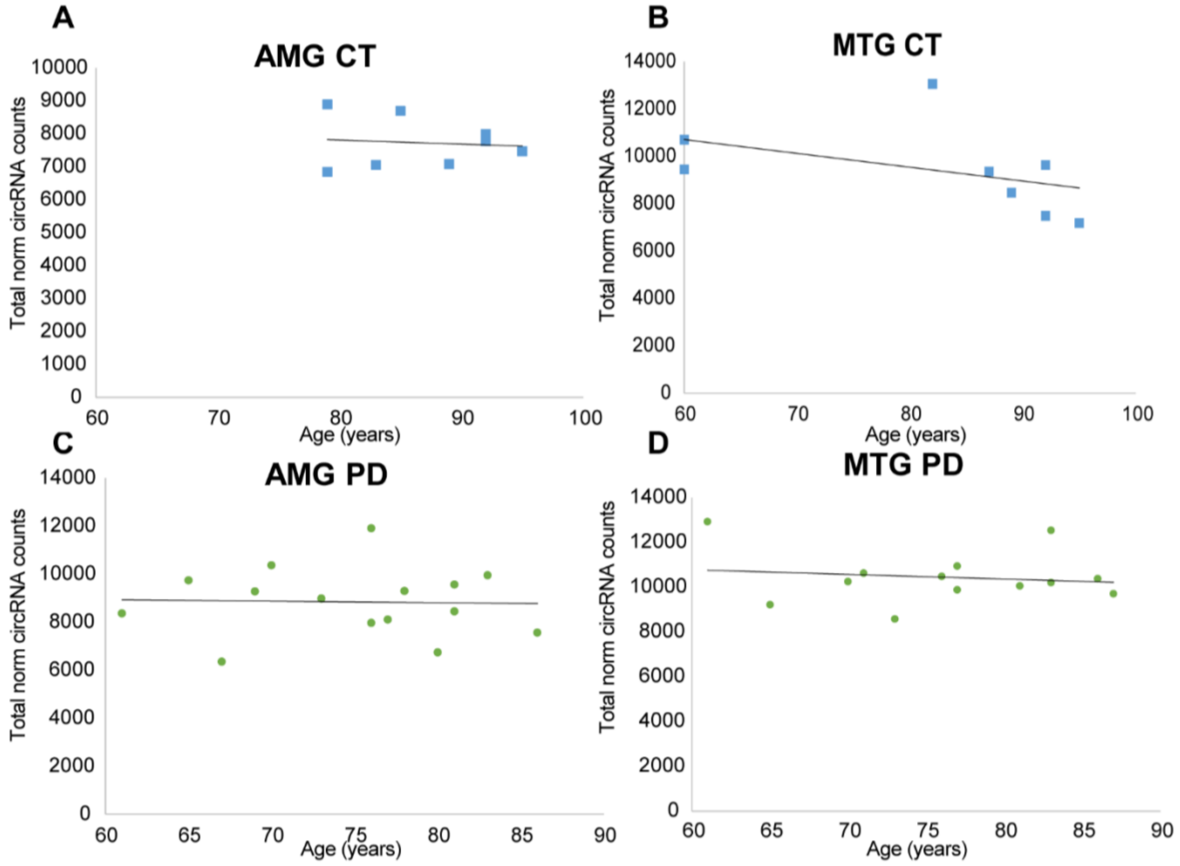

Appendix figure S2: **No positive correlation between age and circRNA expression in MTG or Amygdala control and PD samples:**

(A-D) no correlation in control or PD samples of Amygdala or MTG (Correlation test p value p=0.81 and p=0.91 for CT and PD AMG and p=0.27 and 0.65 for CT and PD in MTG), n=8 for AMG CT, 8 for MTG CT, 15 for AMG PD and 13 for MTG PD.

Appendix figure S3: CircSLC8A1 expression in differentiating ES cells, AgoIP validation and miR-128 levels in SN and under oxidation

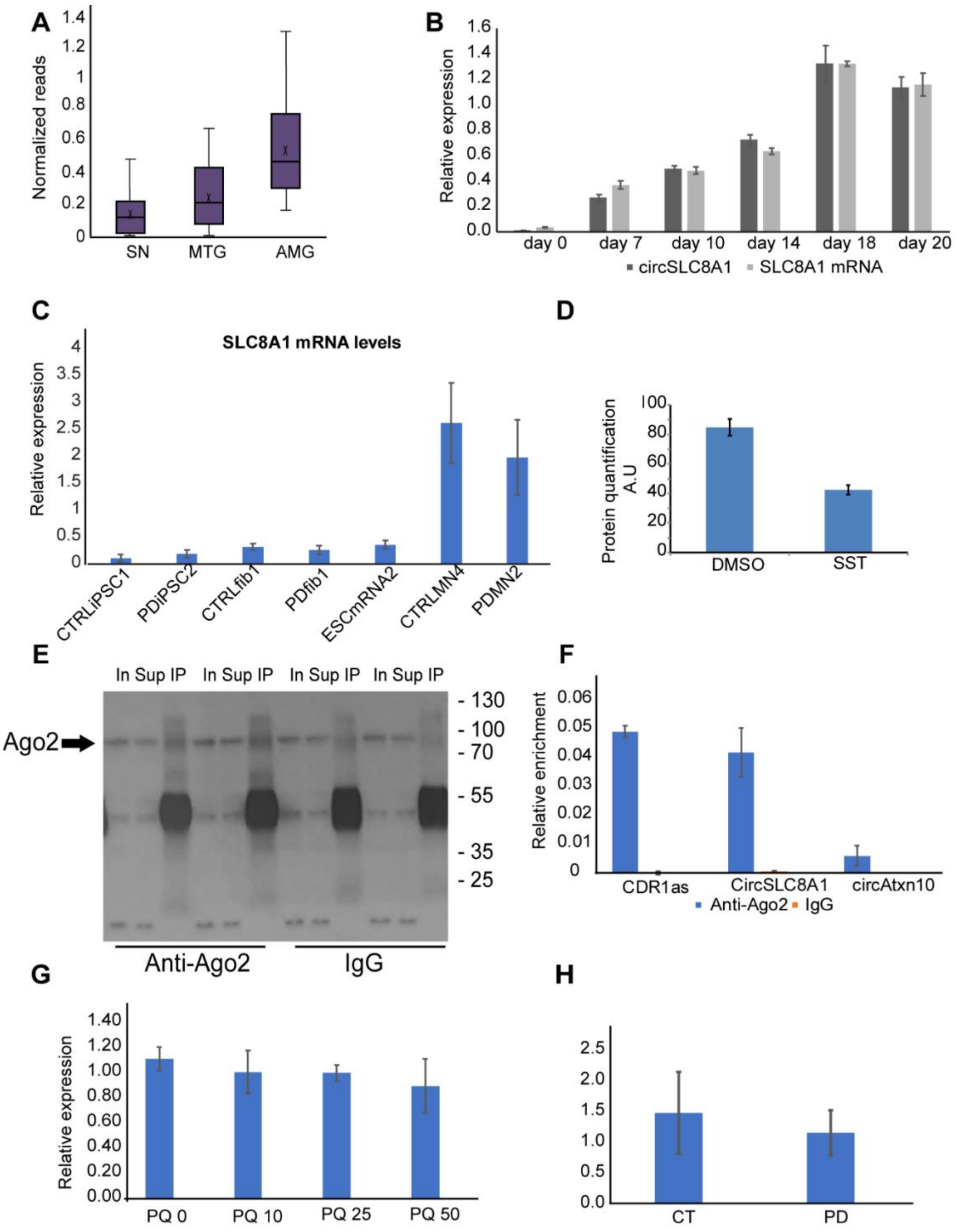

Appendix figure S3: CircSLC8A1 expression in differentiating ES cells, AgoIP validation and miR-128 levels in SN and under oxidation

(A) circSLC8A1 levels in the three testes brain regions. n=8 for Amygdala control, 15 for Amygdala PD, 8 for MTG control and 13 for MTG PD, 10 for SN control and 15 for SN PD. The box is drawn from Q1 to Q3 with a horizontal line drawn in the middle to denote the median and x marks the average. Whiskers mark minimum or maximum values.
(B) circRNA abundance (left) and SLC8A1 mRNA expression (right) in differentiating human ES cells. n=2 biological replicas for each condition. Data presented as mean ± SD.
(C) published datasets show upregulated linear SLC8A1 expression in iPSCs differentiation from healthy and PD fibroblasts into neurons, with in no significant difference in SLC8A1 levels between control and PD samples. n=5 for control fibroblasts, 9 for PD fibroblasts, 6 Control iPSC, 7 PD iPSC, 2 ESCs, 5 Control motor neurons and 6 PD motor neurons. p values calculated by T-test. Data presented as mean ± SD.
(D) quantification of IHC staining using anti-SLC8A1 in SH-SY cells. 10 randomly selected regions of interest (ROIs) from each slide were imaged taking a z-stack of 20um depth to cover the entire area of the cells, 84 cells were analyzed for DMSO and 87 for STT. Data presented as mean ± SD.
(E) WB of Ago2-Immunoprecipitation or IgG control from SHSY cells: Input (In), Sup and pellet (IP). black arrow marks Ago2 band.
(F) qPCR of Ago2-bound RNA and IgG negative control showing circSLC8A1, circCDR1as as a positive control and circATXN10 as negative control. n=3 biological replicas for each condition, Data presented as mean ± SD.
(G) miR-128 levels expression in SHSY cells treated with PQ. n=3 biological replicas for each condition. Data presented as mean ± SD.
(H) miR-128 levels expression in SN of control and PD brains. n=12 for control SN and 17 for PD SN. Data presented as mean ± SD.
