# Supplementary material for: A Parkinson's disease CircRNAs Resource reveals a link between circSLC8A1 and oxidative stress
Source: EMBO Mol Med. 2020 Jul 26;12(9):e11942. doi: 10.15252/emmm.201911942 (PMC7507321; doi:10.15252/emmm.201911942)
Supplement: Supplementary file 2 — Expanded View Figures PDF [file EMMM-12-e11942-s002.pdf]

Expanded View Figures

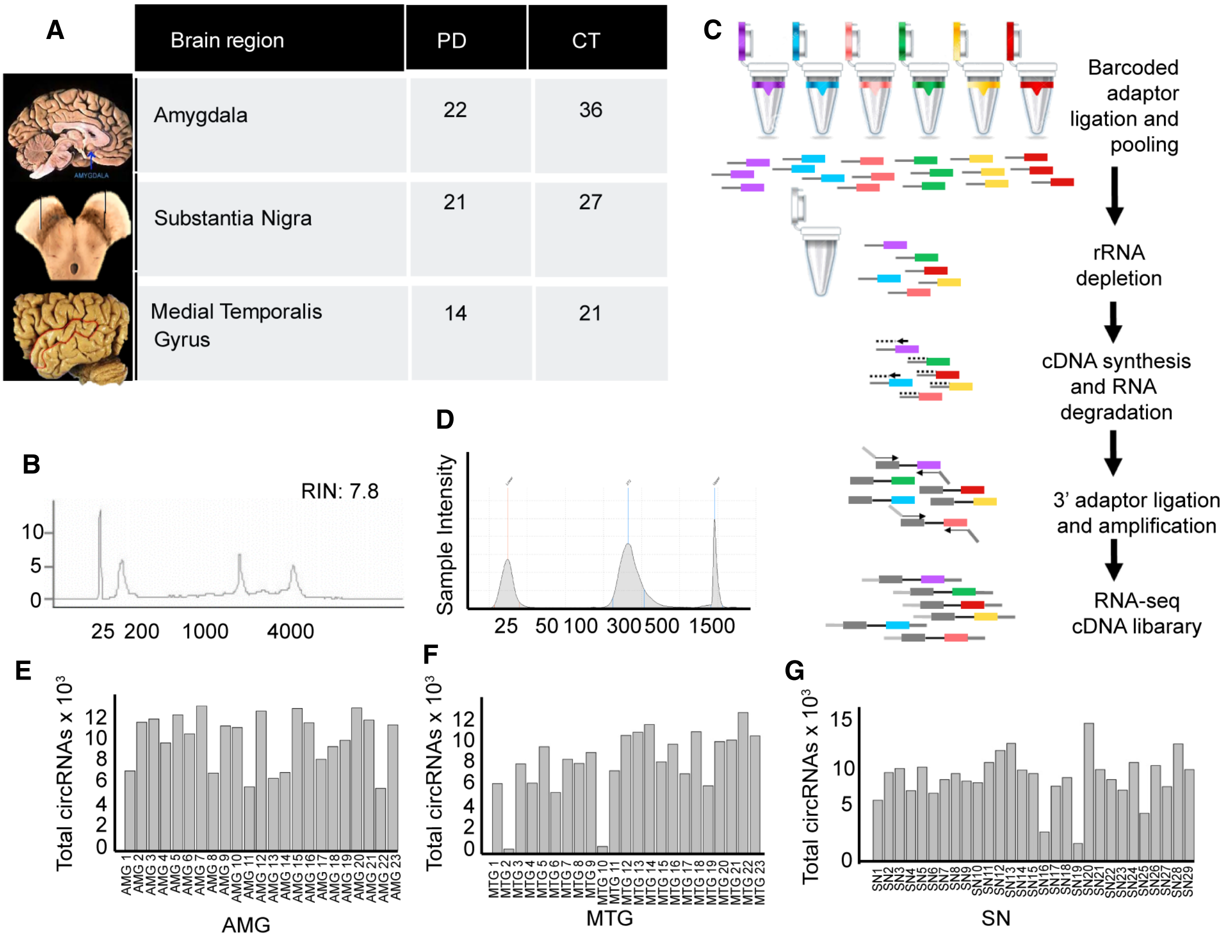

H

Tissue	CT	PD
Amygdala	8	15
MTG	8	12
SN	11	15
total	27	42

I

	SN	Amygdala	MTG
Total reads	1,511,186,328	1,190,791,418	1,141,282,032
Number of samples	29	23	23
Average reads per sample	52,109,873.38	51,773,539.91	49,620,957.91
Total circRNAs detected	367,504	282,177	268,914
Average circRNAs per sample	12,673	12,269	11,692

Figure EV1.

© 2020 The Authors

◀ **Figure EV1. Tissues retrieved and experimental in-house RNA sequencing methodology.**

A Brain region origin of tissues retrieved from the NBB detailing each brain area.
B Average RIN plot of RNA prepared from the frozen tissues.
C Library preparation key steps (see details in Materials and Methods).
D Library plot in a TapeStation run.
E–G Number of circRNAs detected in each sample of all 3 regions. Samples with < 5,000 circRNAs were removed from the analysis.
H Number of samples from PD and control from each tissue that were included in the analysis.
I Number of total reads and circRNAs from each tissue.

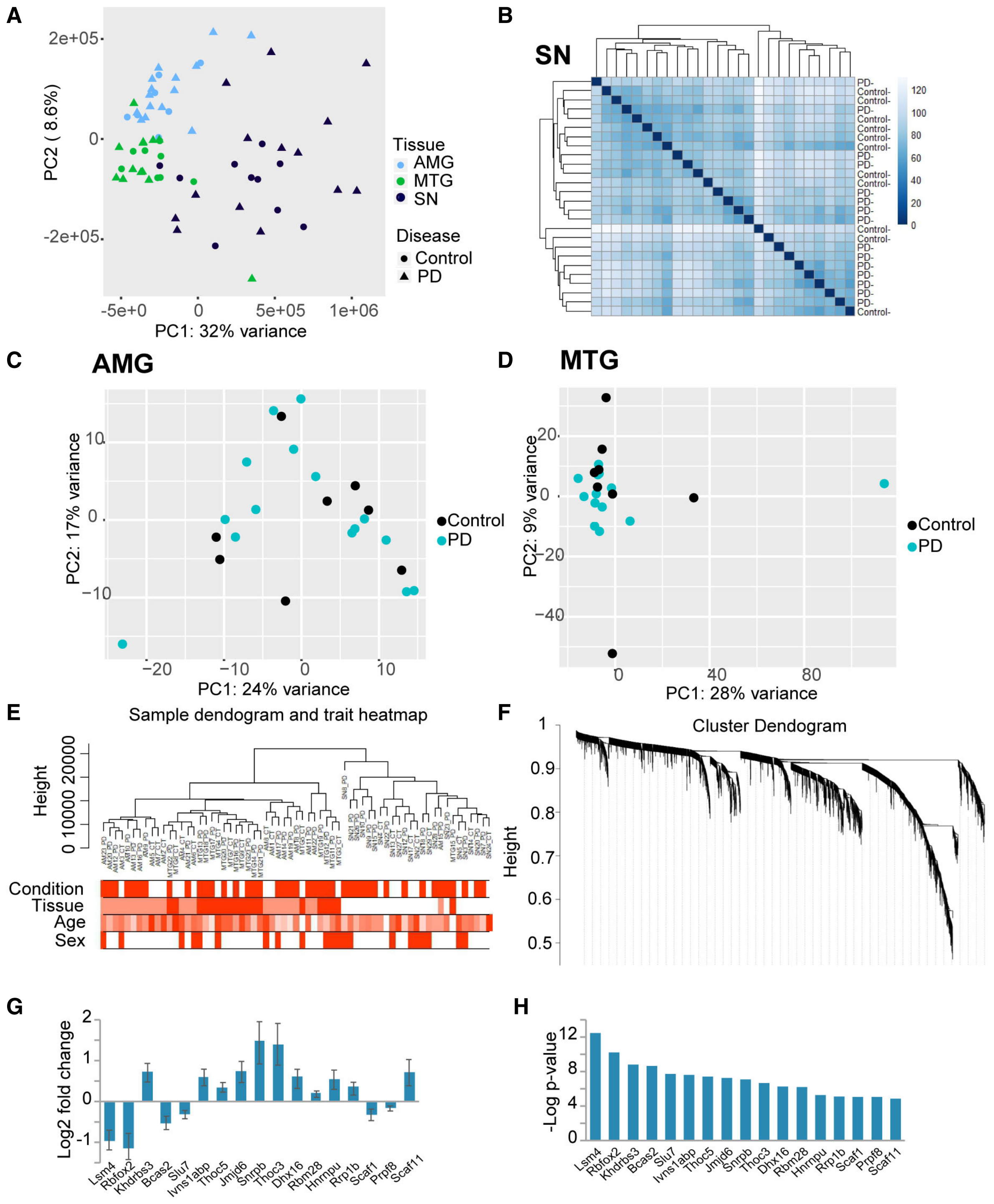

Figure EV2.

© 2020 The Authors

Figure EV2. PCA analysis of all tissues and of PD vs control samples, WGCNA analysis QC, clustering of the samples and modules.

A PCA of all mRNA samples from all tissues.

B Substantia nigra sample heatmap, relative expression of each gene is represented by colors, marked by the color key: Reduction in expression is represented by light green and elevation is represented by blue.

C, D PCA plot of control vs PD samples in the amygdala and MTG.

E Sample dendrogram showing sample clustering and the different classification patterns of external traits for each sample.

F Cluster dendrogram of all genes.

G, H $\log_2$ fold change and $-\log P$ value of known DE splicing factors in PD SN vs control SN. $n = 10$ for SN control and 15 for SN PD. Data presented as mean $\pm$ SD, Walt test (DEseq2 analysis).

© 2020 The Authors

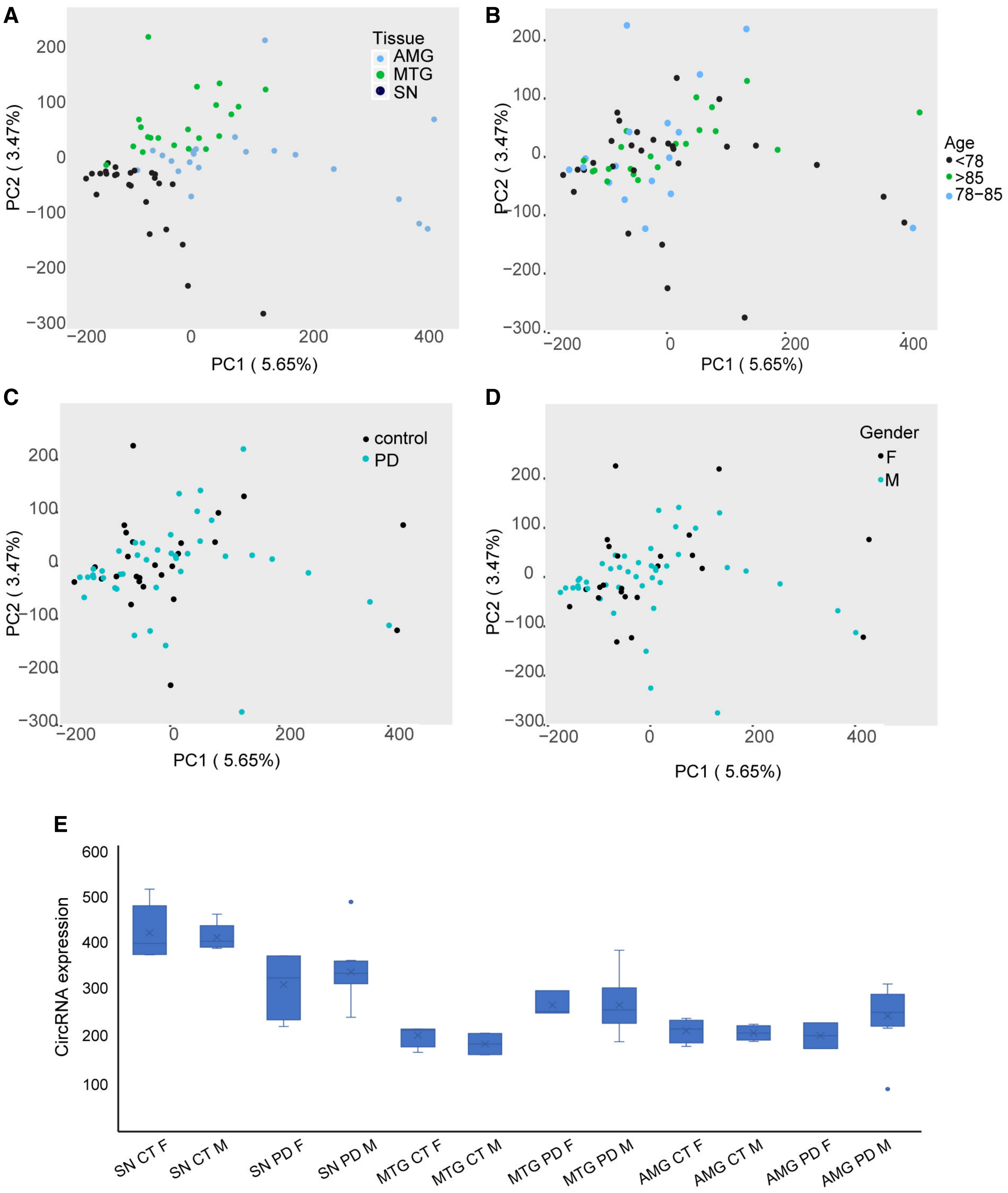

Figure EV3. PCA analysis of circRNA profile.

A–D PCA analysis of circRNA profiles in samples according to specific tissues, age, condition, and gender.

E CircRNA abundance does not change in a gender-specific manner in the tested tissues. $n = 23$ for females and 46 for males. The box is drawn from Q1 to Q3 with a horizontal line drawn in the middle to denote the median and x marks the average. Whiskers mark minimum or maximum values.

 © 2020 The Authors

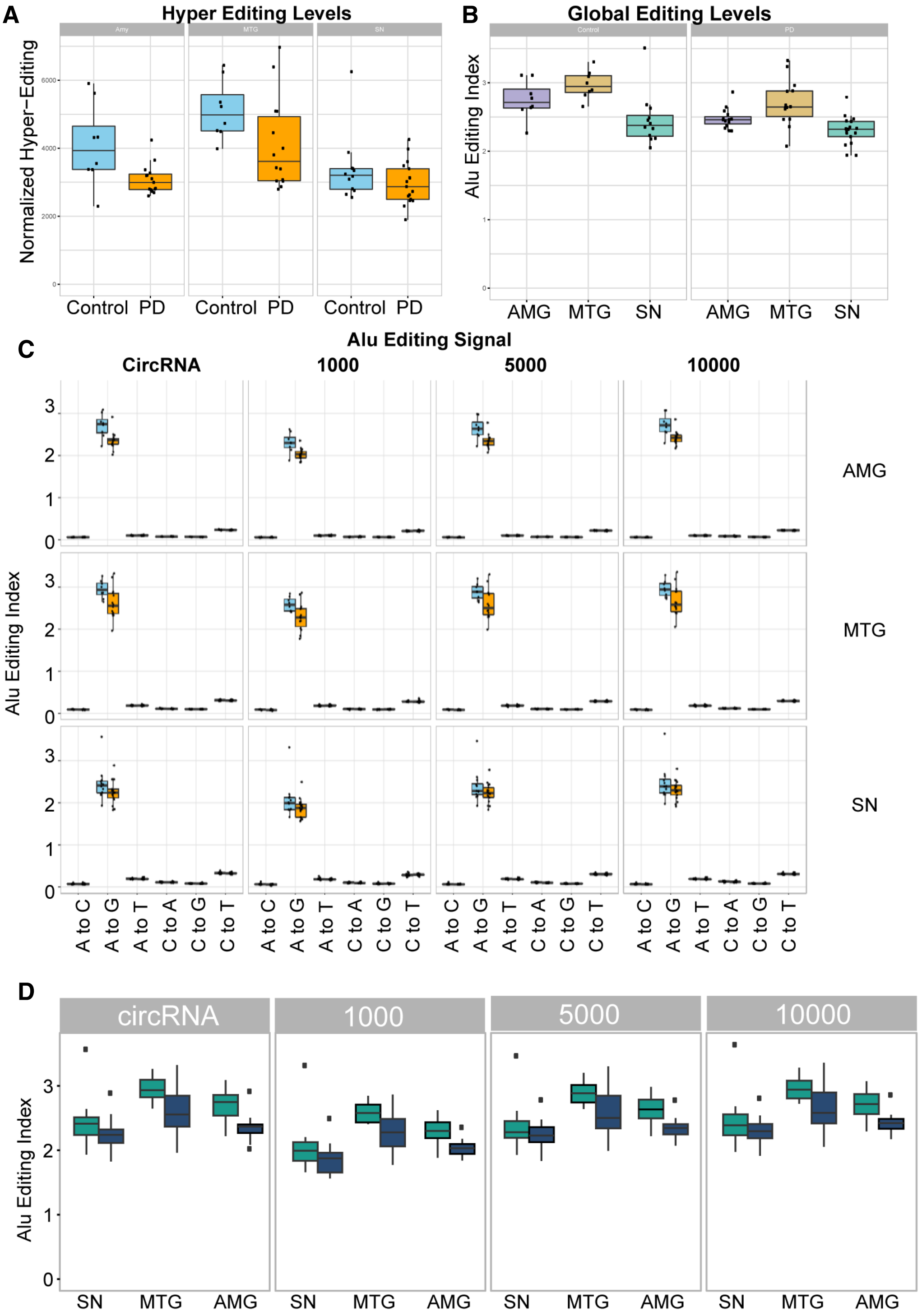
A
Hyper Editing Levels
Normalized Hyper−Editing
Control PD
Control PD
Control PD
B
Global Editing Levels
Alu Editing Index
AMG MTG SN
AMG MTG SN
C
Alu Editing Signal
CircRNA
1000
5000
10000
AMG
MTG
SN
Alu Editing Index
A to C
A to G
A to T
C to A
C to G
C to T
D
circRNA
1000
5000
10000
Alu Editing Index
SN MTG AMG

Figure EV4.

Figure EV4. RNA Editing in PD sample and controls.

A Hyper-editing levels in all 3 tissues, PD, and controls, $n = 8$ for amygdala control, 15 for amygdala PD, 8 for MTG control and 13 for MTG PD, 10 for SN control and 15 for SN PD. The box is drawn from Q1 to Q3 with a horizontal line drawn in the middle to denote the median and x marks the average. Whiskers mark minimum or maximum values.

B Global editing levels measured by *Alu* editing index comparing the three different tissues in PD or control, $n = 8$ for amygdala control, 15 for amygdala PD, 8 for MTG control and 13 for MTG PD, 10 for SN control and 15 for SN PD. The box is drawn from Q1 to Q3 with a horizontal line drawn in the middle to denote the median and x marks the average. Whiskers mark minimum or maximum values.

C *Alu* editing index showing that A-to-G mismatch is the most dominant, as other mismatches are a result of noise or other biological mechanisms and should be much lower than the A-to-I (viewed as A-to-G) RNA editing signal, $n = 8$ for amygdala control, 15 for amygdala PD, 8 for MTG control and 13 for MTG PD, 10 for SN control and 15 for SN PD. The box is drawn from Q1 to Q3 with a horizontal line drawn in the middle to denote the median and x marks the average. Whiskers mark minimum or maximum values.

D *Alu* editing index in circRNAs and flanking introns, in 3 different intron windows: 1,000, 5,000, and 10,000 nucleotides, $n = 8$ for amygdala control, 15 for amygdala PD, 8 for MTG control and 13 for MTG PD, 10 for SN control and 15 for SN PD. The box is drawn from Q1 to Q3 with a horizontal line drawn in the middle to denote the median and x marks the average. Whiskers mark minimum or maximum values.

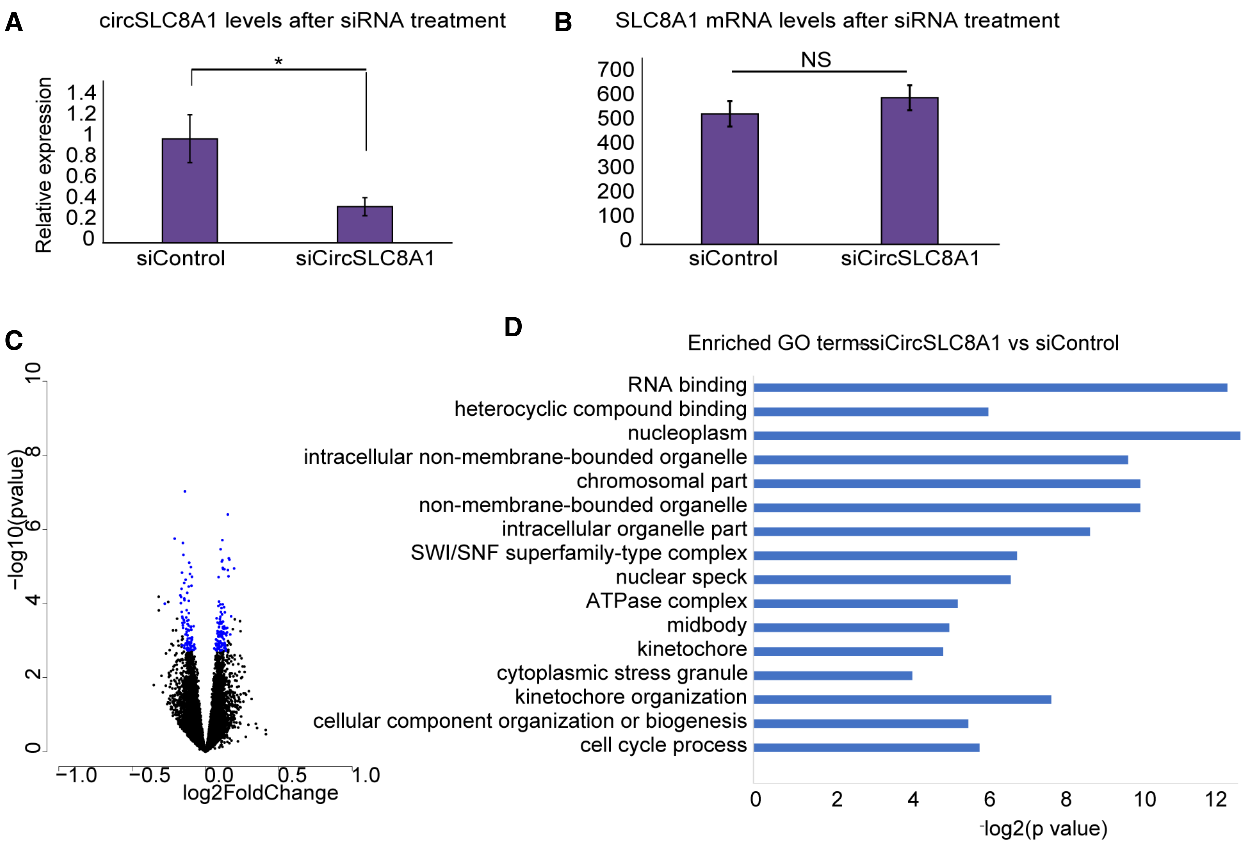

Figure EV5. Gene expression of circSLC8A1 knockdown.

A circSLC8A1 expression after siRNA treatment (*t*-test $*P = 0.0075$), $n = 3$ biological replicas for each condition. Data presented as mean ± SD.

B siRNA targeting circSLC8A1 does not affect SLC8A1 mRNA expression after siRNA treatment (*t*-test $P > 0.05$). $n = 6$ for each condition. Data presented as mean ± SD.

C Volcano plot of gene expression profile of siCircSLC8A1 compared to siControl, blue dots indicate corrected $P < 0.05$, FDR correction of Wald test (DEseq2 analysis).

D Enriched Go terms of DE genes in siCircSLC8A1 compared to siControl analysis, FDR correction Wald test (DEseq2 analysis).

© 2020 The Authors
